# Metabolite Profiling and Microbial Community of Traditional Meju Show Primary and Secondary Metabolite Differences Correlated with Antioxidant Activities

**DOI:** 10.4014/jmb.2007.07026

**Published:** 2020-09-02

**Authors:** Da Hye Song, Byung Hee Chun, Sunmin Lee, Chagam Koteswara Reddy, Che Ok Jeon, Choong Hwan Lee

**Affiliations:** 1 Department of Bioscience and Biotechnology, Konkuk University, Seoul 05029, Republic of Korea; 2 Department of Life Science, Chung-Ang University, Seoul 06974, Republic of Korea; 3 Department of Systems Biotechnology, Konkuk University, Seoul 05029, Republic of Korea; 4 Research Institute for Bioactive-Metabolome Network, Konkuk University, Seoul 05029, Republic of Korea

**Keywords:** Fermented soybean paste, metabolomics, microbial community, antioxidant activity

## Abstract

Meju, a type of fermented soybean paste, is used as a starter in the preparation of various Korean traditional soybean-based foods. In this study, we performed Illumina-MiSeq paired-end sequencing for microbial communities and mass spectrometry analysis for metabolite profiling to investigate the differences between 11 traditional meju products from different regions across Korea. Even though the bacterial and fungal communities showed remarkable variety, major genera including *Bacillus*, *Enterococcus*, *Variovorax*, *Pediococcus*, *Weissella*, and *Aspergillus* were detected in every sample of meju. The metabolite profile patterns of the 11 samples were clustered into two main groups: group I (M1-5) and group II (M6-11). The metabolite analysis indicated a relatively higher amino acid content in group I, while group II exhibited higher isoflavone, soyasaponin, and lysophospholipid contents. The bioactivity analysis proved that the ABTS (2,2'-azino-bis (3- ethylbenzothiazoline-6-sulfonic acid)) radical-scavenging activity was higher in group II and the FRAP (ferric reducing antioxidant power) activity was higher in group I. The correlation analysis revealed that the ABTS activity was isoflavonoid, lipid, and soyasaponin related, whereas the FRAP activity was amino acid and flavonoid related. These results suggest that the antioxidant activities of meju are critically influenced by the microbiome and metabolite dynamics.

## Introduction

Meju is a source of natural microorganisms which produce various enzymes that degrade the macromolecules in soybean [1], the raw material of meju. Soybeans are known to vary greatly depending on region, climate, and soil, and studies have shown that metabolic differences in soybean are caused by the diversity of soybean genes [2, 3]. The fermentation process involved in crushing, steaming and shaping soybean into bricks of meju, is the most important step in producing other fermented soy foods, especially doenjang (thick bean paste) and ganjang (soy sauce), because the small metabolites that contribute to the nutritional qualities of these foods are produced during meju fermentation [1]. Traditional fermented soybean-based foods such as doenjang, cheonggukjang (fast-fermented soybean paste), and ganjang have been reported to exhibit a variety of functional properties, viz. antioxidant, anti-microbial, anti-diabetic, anti-cholesterol, anti-cancer, anti-genotoxic, and anti-inflammatory effects [4]. Commercial doenjang is made using slightly different procedures and the finished product varies depending on the shape or size of the meju, and salt concentration [5, 6]. In meju, bacteria and fungi are responsible during fermentation for the hydrolysis of key macromolecular components, including proteins, lipids, carbohydrates, and flavonoid glycosides. These microorganisms are also associated with the production of various metabolites, such as amino acids, organic acids, active metabolites, and aglycone, which contribute to the nutritional value of soy products. Fermented soybean products are affected by the variety of microorganisms, which in turn determines their characteristic taste and flavor [7]. In particular, *Bacillus* and *Aspergillus* are known as the predominant species of fermented soybean foods such as meju [8, 9].

In recent years, metabolomic analysis has been adopted to compensate for the limitations of chromatographic analytical methods as a useful approach for acquiring high-throughput measurements of the metabolites present in cells, tissues, and biofluids. Metabolomics enables the comprehensive analysis of metabolites, making it a valuable tool in food science for the assessment of food quality and component analysis [10]. Previous studies applied a metabolic approach to investigate the metabolic changes during doenjang processing [11] and aging [12], contributing to food quality control standards. Metabolites, including organic acids (such as amino and fatty acids) and sugars, reportedly contribute to the discrimination of samples subjected to different fermentation processes.

In this study, we investigated the component diversity of traditional meju manufactured in local markets. Furthermore, we identified metabolites related to the antioxidant activity of meju using a correlation assay to serve as a quality assessment for the differences in each sample.

## Materials and Methods

### Chemicals and Reagents

HPLC-grade solvents including methanol, water, and acetonitrile were purchased from Fisher Scientific (USA). Standard compounds and analytical grade reagents used in this study were purchased from Sigma Chemical Co.(USA).

### Preparation of Samples

In this work, we used 11 meju samples in triplicate. The products were purchased from different local traditional markets located in different regions of Korea (Table 1). Initially, three meju bricks were collected from each manufacturer and ground using a mixer. For microbial community analysis, a 200 mg sample was taken from each meju brick, combined and mixed well, and stored at −80°C until analysis.

For metabolite analysis, the meju samples were freeze dried and then pulverized using a mortar and pestle. Then, each sample (200 mg) was extracted with aqueous methanol (1 ml, 80%), using an MM400 mixer mill (Retsch Haan, Germany) at a frequency of 30 s^−1^ for 10 min, followed by sonication at 4°C for 1 min. After sonication, the sample dispersion was centrifuged at 17,000 ×*g* for 10 min at 4°C, and the resultant supernatant was filtered through a 0.22-μm Milex (Merck Millipore, USA). Then, the soluble filtrates were dried using a speed-vacuum concentrator (Biotron; Korea) and stored at ‒20°C for further analysis.

### Illumina-MiSeq Paired-End Sequencing for Bacterial and Fungal Community Analysis

Total genomic DNA was extracted from the pooled meju samples using a FastDNA Spin Kit (MPbio, USA), according to the manufacturer’s instructions. Two primer sets, 341F (5′-adaptor-CCT ACG GGN GGC WGC AG-3′)/805R (5′-adaptor-GAC TAC HVG GGT ATC TAA TCC-3′) and 3271-ITS2F (5′-adaptor- CAR CAA YGG ATC TCT TGG-3′)/3271-ITS2R (5′-adaptor- GAT ATG CTT AAG TTC AGC GGG T-3′) were used for the amplification of bacterial 16S rRNA (V3–V4 variable regions) and the ITS2 region of fungal rRNA genes, respectively. All the PCR amplifications were performed in a C1000 Thermal Cycler (Bio-Rad, USA) in a 50-μl reaction volume containing Taq polymerase mixture, 1 μl genomic DNA, and 20 pmol of each primer with the following cycling settings: 94°C for 5 min (1 cycle); 94°C for 45 sec, 55°C for 45 sec, and 72°C for 1 min (30 cycles); and 72°C for 10 min (1 cycle). The PCR amplicons were purified using a PCR purification kit (Solgent, Korea) and their concentrations were carefully measured with an ELISA reader equipped with a Take3 Multi-Volume Plate (SynergyMx; BioTek, USA). A composite sample was prepared by pooling equal amounts of purified PCR products and sequenced using an Illumina Miseq platform (Roche, Germany) at Macrogen (Korea). The obtained Illumina Miseq sequencing reads were sorted into individual meju samples according to their barcode sequences. The barcode and adapter sequences were removed using the Scythe program (v.0.994, https://github.com/vsbuffalo/scythe). Bacterial and fungal communities in each meju sample were analyzed using the Qiime2 plugin software package (v.2020.2, https://qiime2.org/) according to the procedure described previously [13].

### GC-TOF-MS Analysis

Prior to the gas chromatography time-of-flight mass spectrometry (GC-TOF-MS) analysis, each dried sample extract was subjected to two steps of a derivatization reaction, following a method described by Lee *et al*. [14]. First, the oximation was performed by adding 50 μl of methoxyamine hydrochloride in pyridine (20 mg/ml) to each sample extract and incubating the reaction at 30°C for 90 min. Next, the silylation was achieved by adding 50 μl of N-methyl-N-(trimethylsilyl) trifluoroacetamide (MSTFA) followed by reaction incubation at 37°C for 30 min. The derivatized samples were filtered using a Millex GP 0.22-μm filter prior to instrument analysis and the final concentration of derivatized sample was 20 mg/ml.

GC-TOF-MS analysis was performed using an Agilent 7890A gas chromatography system (Agilent Technologies, USA) equipped with an Agilent 7693 Autosampler and the Pegasus high-throughput (HT)-TOF-MS program (Leco Corp., USA). The metabolites were separated using an Rtx-5MS column (30 m × 0.25 mm; 0.25 μm; Restek Corp. USA) and the operational parameters were adapted from a study by Lee *et al*. [14].

### UHPLC-Orbitrap-MS/MS Analysis

For ultra-high-performance liquid chromatography quadrupole orbitrap ion trap tandem mass spectrometry (UHPLC-Q-orbitrap-MS/MS) analysis, each dried sample (20 mg/ml) was dissolved in aqueous methanol (80%) and used. UHPLC-Orbitrap-MS/MS analysis was performed using a UHPLC system equipped with a Vanquish Binary Pump H System (Thermo Fisher Scientific, USA) coupled with auto-sampler and column compartment. The chromatographic separation was performed on a Phenomenex KINETEX C18 column (100 mm × 2.1 mm, 1.7 μm; USA) and the operational parameters were adapted from a study by Lee *et al*. [14].

### Data Processing and Multivariate Statistical Analysis

The raw data files from GC-TOF-MS and UHPLC-Q-Orbitrap-MS/MS were converted into computable document form (.cdf) format using Chroma TOF software v.4.44 (LECO Co.) and Xcalibur v.2.2 (Thermo Fisher Scientific). After conversion, the software MetAlign (http://www.metalign.nl) was used for preprocessing of netCDF data to acquire peak extraction, retention time correction, peak intensity normalization, and accurate masses. Subsequent data were transferred to an Excel sheet and multivariate statistical analysis was executed by SIMCA-P^+^ v.12.0 (Umetrics, Sweden). Furthermore, both unsupervised principal component analysis (PCA) and supervised partial least squares discriminant analysis (PLS-DA) were performed to compare the different metabolites of the samples. The discriminant metabolites were selected based on variable importance in projection value (VIP > 0.7) and tested for significance at a *p*-value (*p* < 0.05). The selected metabolites were identified by comparing their retention time, mass spectrum, and mass fragment patterns with standard compounds, references, in-house library data, and various commercial databases, such as the National Institutes of Standards and Technology (NIST) Library (v.2.0, 2011, FairCom, USA), the Dictionary of Natural Products (v.16:2, 2007, Chapman and Hall, USA), Wiley 8, BioCyc Database Collection (https://biocyc.org/), and the Human Metabolome Database (HMDB; http://www.hmdb.ca/). The significances (*p* < 0.05) were tested by one-way ANOVA using Statistica (v.7.0, StatSoft Inc., USA). The Pearson’s correlation coefficient between the metabolites and the corresponding phenotype was performed using Predictive Analytics Software (PASW) Statistics 18 software (SPSS Inc., USA).

### Antioxidant Activity Analysis

ABTS (2,2'-azino-bis (3-ethylbenzothiazoline-6-sulfonic acid), FRAP (ferric reducing antioxidant power), and DPPH (2,2-diphenyl-1-picryl-hydrazyl) radical scavenging assays were performed to measure the in vitro antioxidant activities of the different meju samples (20 mg/ml^-1^ methanol), following a method reported by Lee *et al*. [14]. The antioxidant results are represented as Trolox equivalent antioxidant capacity (TEAC) concentration (mM).

### Total Phenolic Content and Total Flavonoid Content

Total phenolic content (TPC) and total flavonoid content (TFC) in the 11 meju samples were measured, following a method reported previously by Son *et al*. [15]. TPC assay results were expressed in terms of gallic acid equivalents of the activity (μg/ml) and the TFC assay results were expressed as naringin equivalent activity concentrations (μg/ml).

## Results and Discussion

### Microbiome and Metabolomic Profiling of the 11 Meju Samples Manufactured in Different Local Markets

Numerous studies have previously investigated the changes in the microbial community and metabolism in *meju* [16]. Metabolite profiling was used to analyze the changes in the metabolite states of fermented soy products, including meju [16-18], doenjang [19], cheonggukjang [20], and gochujang (red pepper paste) [21, 22]. Despite being soy-derived fermented food products, a single ingredient, meju, differed metabolically in each sample. Since there was a study demonstrating that the distribution of microorganisms in each meju sample affected its metabolite content, we hypothesized that the different metabolites in the 11 meju samples were also diverse. In this study, we employed MS-based techniques combined with multivariate analysis to demonstrate a comprehensive metabolite variety in 11 traditional meju samples and their microbial communities to assess the comprehensive metabolic and biochemical events underlying meju manufacturing in different local markets.

### Bacterial and Fungal Community of the 11 Meju Samples

**Fig. 1:**
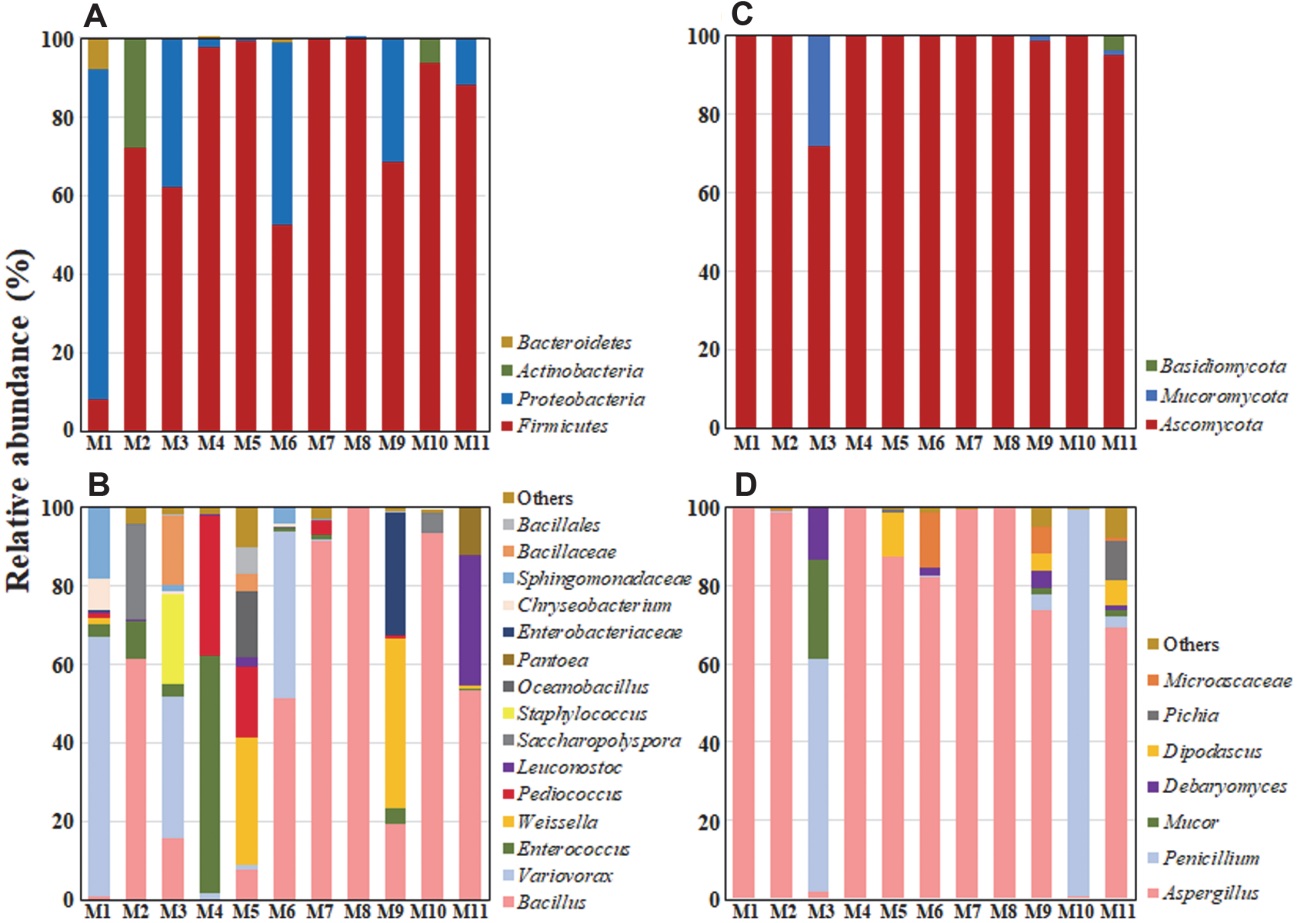
Relative abundances of bacteria (A and C) and fungi (B and D) present in the 11 meju samples at the phylum (A and B) and genus (C and D) levels. “Others” consists of bacterial and fungal groups with < 4% relative abundance in all samples.

Illumina Miseq sequencing reads of bacterial 16S rRNA and fungal ITS2 genes in 11 meju samples were categorized at the phylum and genus levels to explore their microbial communities (Fig. 1). At the phylum level of bacteria, Firmicutes was predominant in most meju samples except for M1 sample and Proteobacteria was also identified at high abundances. At the phylum level of fungi, Ascomycota predominated in all meju samples. The genus level analysis of bacteria showed that bacterial communities varied significantly depending on the meju samples, indicating that fermentation conditions for each of the 11 samples might be different (Table 1). However, *Bacillus*, *Enterococcus*, *Variovorax*, *Pediococcus*, and *Weissella* were generally identified as major bacterial genera. *Bacillus* was the most common and dominant bacterial genus in the 11 meju samples. The average ratio of *Bacillus* was the highest in M2, M6, M7, M8, M10, and M11. The *Bacillus*-dominant samples showed a trend of clustering in PLS-DA (Fig. 3A). The *Bacillus* species produce enzymes such as amylase, which breaks down glucose polysaccharides during endocytosis, and protease, which hydrolyzes peptide bonds in proteins or peptides during the meju fermentation [23]. The genus level analysis of fungi showed that *Aspergillus* was the predominant fungal group present in most meju samples, except for M3 and M10, but *Penicillium* was also identified predominantly from M3 and M10 samples. Especially, *Aspergillus* species produce enzymes such as proteases and amylase that hydrolyze proteins and polysaccharides in fermented food. Previously, Lee *et al*. [11] described that during soybean fermentation by *Aspergillus* sp., the aglycone and hydroxy-isoflavone contents were increased due to the glucosidase-mediated glycoside hydrolysis. It has also been reported that *Bacillus*, *Variovorax*, *Enterococcus*, *Weissella*, *Aspergillus*, and *Penicillium*, which were relatively abundant in our 11 meju samples, are well-known microorganisms commonly found in soybean-derived fermented foods [8, 24, 25].

### Metabolite Profiling of 11 Meju Samples Manufactured in Different Local Markets

**Fig. 2:**
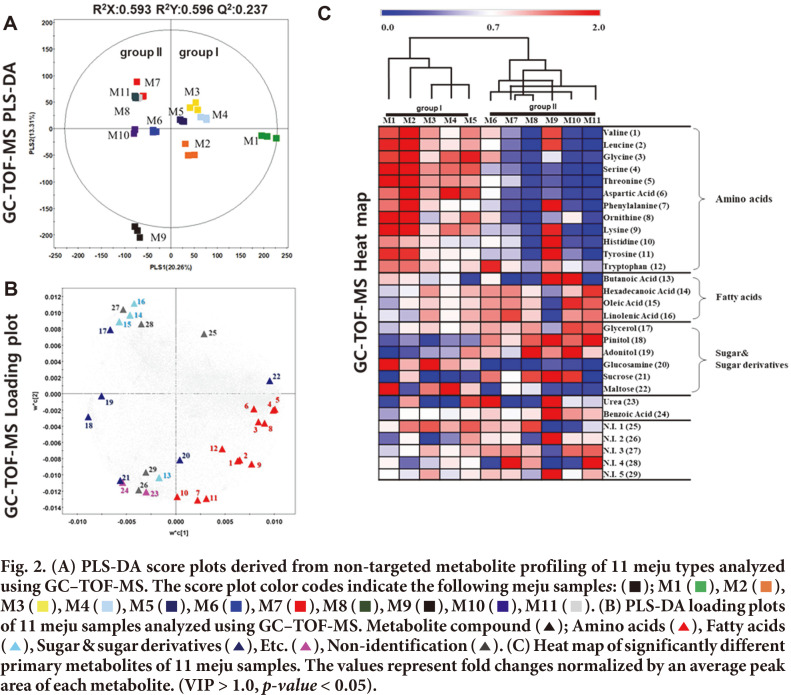


To investigate the metabolite profiles of the 11 meju samples prepared in different local markets, we analyzed the samples using GC-TOF-MS and UHPLC-Orbitrap-MS/MS, followed by multivariate statistical analysis of the corresponding datasets. Multivariate analyses of the aligned datasets demonstrated a distinct pattern in the unsupervised PCA and supervised PLS-DA models. The PLS-DA score plot based on GC-TOF-MS analysis presented a total variance of 30.57% (PLS1, 20.26%; PLS2, 10.31%) with statistical variants 0.593 (R^2^X), 0.596 (R^2^Y), and 0.237 (Q^2^) (Fig. 2A). Furthermore, the PLS-DA analysis demonstrated that the 11 meju samples were clustered into two groups by PLS1, namely group I (M1–5) and group II (M6–11). Similar with the PLS-DA analysis, the PCA score plot (Fig. S1) could also be readily divided into two groups, along the PC1 (20.31%). The GC-TOF-MS analysis identified a total of 24 significantly discriminant metabolites (Table S1). The discriminant metabolites that contributed to the isolation along the PLS1 are also shown in the loading plot (Fig. 2B). The relative abundance of metabolites was compared using a heat map (Fig. 2C). Metabolites exhibiting higher levels in M1 and M2 are valine (**1**), leucine (**2**), glycine (**3**), serine (**4**), threonine (**5**), aspartic acid (**6**), phenylalanine (**7**), ornithine (**8**), lysine (**9**), histidine (**10**), tyrosine (**11**), and tryptophan (**12**). Hexadecenoic acid (**14**), oleic acid (**15**), and linoleic acid (**16**) were abundant in M7, M8, M10 and M11. The contents of urea (**23**) and benzoic acid (**24**) are abundantly identified in M9. In summary, the relative content of amino acids was higher in group I than in group II in the 11 meju sample metabolite heat maps (Fig. 2C). Furthermore, the amino acids threonine, serine, glycine, alanine, and lysine contribute to a sweet taste, while aspartic acid, glutamic acid, and cysteine c ontribute to the umami taste of fermented soybean foods [26]. It is assumed that amino acids affect the taste and aroma of meju [27]. Moreover, fatty acids, such as hexadecenoic acid, oleic acid, and linoleic acid, were detected in other fermented soybean products such as miso [28].

**Fig. 3:**
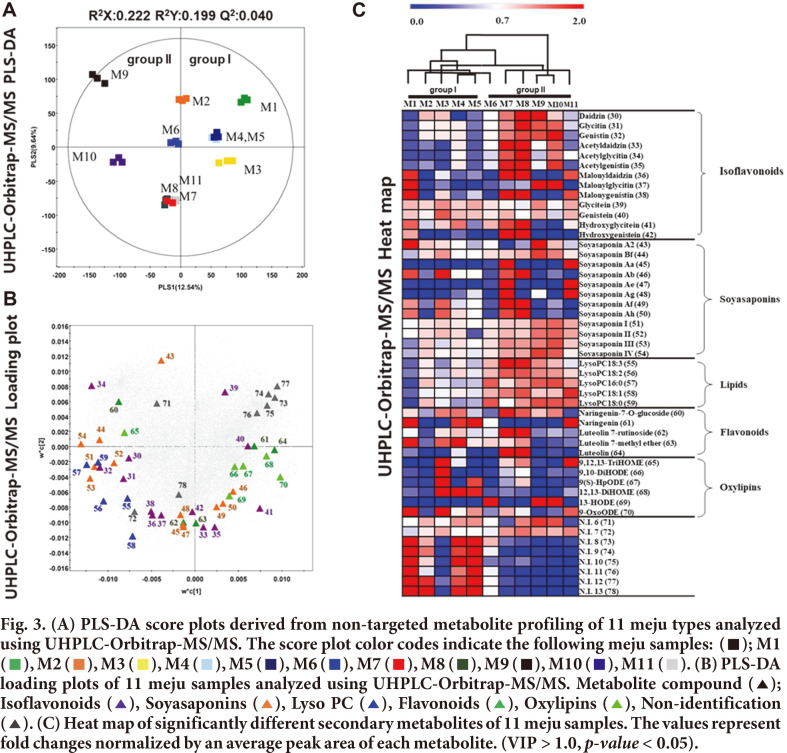


The PLS-DA model, based on 11 meju samples analyzed using UHPLC-Orbitrap-MS/MS, exhibited a pattern similar to that of the GC-TOF-MS analysis. The PLS-DA score plot showed 12.54 % and 9.24 % variance by the PLS1 and PLS2, respectively (Fig. 3A). Group I (M1–5) and group II (M6–11) were separated by PLS1 similar to the results of the GC-TOF-MS analysis (Fig. 2A). M6 in group II was clustered by group. However, it was located in the center of the 11 samples in the PLS-DA plot and the trends were unclear in the case of certain compounds. UHPLC-Orbitrap-MS/MS analysis identified a total of 41 metabolites (Table S2). From the UHPLC-Orbitrap-MS/MS loading plot (Fig. 3B), the daidzin (**30**), glycitin (**31**), genistin (**32**), acetyldaidzin (**33**), acetylgenistin (**35**), malonyldaidzin (**36**), malonylglycitin (**37**), malonylgenistin (**38**), hydroxygenistin (**42**), soyasaponin Bf (**44**), soyasaponin Aa (**45**), soyasaponin Ae (**47**), soyasaponin Ag (**48**), soyasaponin I (**51**), soyasaponin II (**52**), soyasaponin III (**53**), soyasaponin IV (**54**), lysoPC18:3 (**55**), lysoPC18:2 (**56**), lysoPC16:0 (**57**), lysoPC18:1 (**58**), and lysoPC18:0 (**59**) contents were abundant in M7, M8, M10, and M11. The 9,12,13-TriHOME (**65**), 9,10-DiHODE (**66**), 9(**S**)-HpODE (**67**), 12,13-DiHOME (**68**), and 9-OxoODE (**70**) contents were abundant in M3. The N.I. 8 (**73**) and N.I. 12 (**77**) contents were abundant in M1. In the UHPLC-Orbitrap-MS/MS heat map (Fig. 3C), the amounts of secondary metabolites that were abundant for each sample were compared. Isoflavones and soyasaponins are known as characteristic soybean components and one of the main ingredients of doenjang [11, 29, 30]. Isoflavones are phytoestrogens, similar in structure to 17-β-estradiol with reportedly less active estrogen-like activity than hormones. Usually, most isoflavones exist in the form of glycoconjugates (genistin, daidzin, and glycitin), or acetyl or malonyl derivatives [31]. In particular, the glycoside forms of isoflavones are not absorbed due to their high hydrophilicity and molecular weight, as well as their low estrogenic activity. However, aglycone hydrolysis (genistein, daidzein, and glycitein) increases their bioavailability [32]. In the 11 meju samples, the glycoside form of isoflavones was higher than the aglycone form. The aglycone form of isoflavonoids is present at a higher level in doenjang [33, 34]. The structure of soyasaponin has one or more glycoside moieties in the lipophilic triterpene derivative. Saponins present in various forms are divided into two groups. Group B soyasaponin reportedly exhibits health-promoting properties [35]. Group A soyasaponins (soyasaponin A2, Aa, Ab, Ae, Ag, Ae, and Af) and group B soyasaponins (soyasaponin I, II, III, and IV) were detected in the 11 meju samples. In group B soyasaponins, the DDMP-conjugated soyasaponin is not stable, and it is thus easily converted to non-DDMP soyasaponin, known as soyasaponin I, II, III, IV, and V. Raw soybean mainly consists of DDMP-conjugated soyasaponin, but processed soy products are mainly composed of non-DDMP soyasaponins. In the 11 meju samples, only non-DDMP soyasaponins were detected. Some saponins, including soyasaponins, apparently contribute to the bitter or astringent taste [36] and have different biological effects, including neuroprotective [37] and anti-cancer effects [38]. The specific bioactivity of soybean that is different from other plant-based foods is due to isoflavonoids and soyasaponins, the main secondary metabolites of soy products. Isoflavonoids and soyasaponin, well-known main secondary metabolites of soy products, had higher levels in group II than in group I.

### Antioxidant Activity Assays of 11 Different Meju Samples

**Fig. 4:**
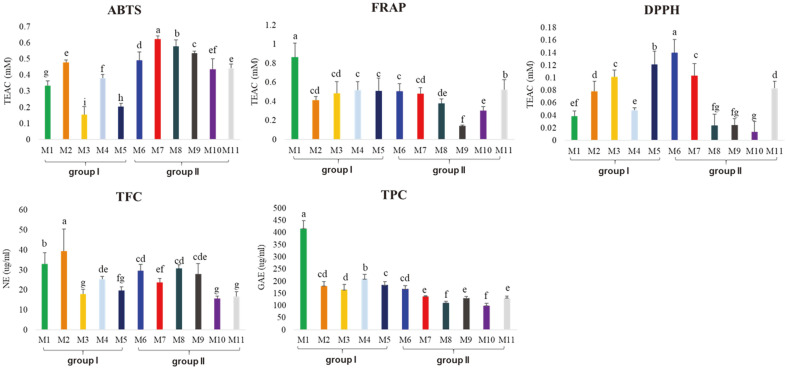
Antioxidant activity analysis using ABTS, FRAP, DPPH, TPC (total phenolic contents), and TFC (total flavonoids contents) of 11 meju samples. Different letters in the bar graph indicate significant differences analyzed by ANOVA followed by Duncan’s new multiple range test.

Antioxidant activity reduces the risk of circulatory system illnesses and cancers by delaying the occurrence of factors associated with disease progression [39]. We observed that the differential metabolomes of the 11 meju samples were directly related to the biochemical functions of antioxidant activity. The antioxidant activity of the 11 meju samples was measured by the following bioassays: ABTS, DPPH, FRAP radical scavenging activity, TPC, and TFC. The results of the antioxidant activity assays revealed that the meju bioactivity was different for each sample (Fig. 4). As with the metabolite analysis results, the 11 meju samples exhibited a trend of differing results for the two groups. The FRAP assay and TPC analysis resulted in higher average values in group I than in group II, while the ABTS assay resulted in higher values in group II than in group I. M6 and M2 were clustered by group, respectively. However, they were in the center in the PLS-DA plot and the trends were unclear in the case of certain bioactivities.

### Correlation between Meju Antioxidant Activities and Metabolite Compositions

**Fig. 5:**
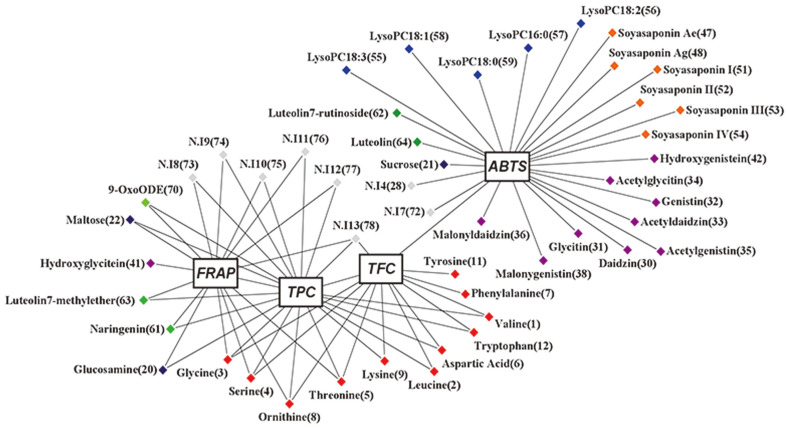
Correlation networks between the metabolites and bioactivity assays (ABTS, DPPH, FRAP, TPC, and TFC). The metabolites were selected based on their Pearson’s correlation value (*r*) > 0.4.

Through metabolite profiling of 11 meju samples (Figs. 2 and 3), it was found that group I and group II were clustered separately. These results were similar to those of the bioactivity analysis. Therefore, we executed a Pearson correlation analysis to confirm the relationship between the metabolite composition and antioxidant activity through a correlation assay (Fig. 5). The correlation network was evaluated for the variables with a Pearson correlation value >0.4. The results of the correlation analysis demonstrated that Daidzin (**30**), glycitin (**31**), genistin (**32**), malonyldaidzin (**33**), malonygenistin (**35**), acetyldaidzin (**36**), acetylglycitin (**37**), acetylgenistin (**38**), hydroxygenistein (**42**), soyasaponin Ae (**47**), soyasaponin Ag (**48**), soyasaponin I (**51**), soyasaponin II (**52**), soyasaponin III (**53**), soyasaponin IV (**54**), lysoPC18:3 (**55**), lysoPC18:2 (**56**), lysoPC16:0 (**57**), lysoPC18:1 (**58**), lysoPC18:0 (**59**), luteolin-7-rutinoside (**62**), luteolin (**64**), N.I. 4 (**28**), N.I. 7 (**72**), sucrose (**21**), and TFC showed strong positive correlations with the ABTS antioxidant activity. In group II, high-content metabolites were linked to ABTS activity. Furthermore, the ABTS activity in group II showed high values due to the structural properties of the correlated metabolites. Flavonoids and isoflavonoids with a B ring structure are well-known antioxidants [40, 41]. A correlation was noted between TFC and ABTS results. Group A and group B saponins are also known to contribute to free radical scavenging [42]. The results of the TFC, TPC, and FRAP antioxidant activity analyses correlated, connecting glycine (**3**), serine (**4**), threonine (**5**), ornithine (**8**), and N.I. 13 (**78**). However, maltose (**22**), glucosamine (**20**), Naringenin (**61**), Luteolin-7-methyl ether (**63**), N.I. 8 (**73**), N.I. 9 (**74**), N.I. 10 (**75**), N.I. 11 (**76**), N.I. 12 (**77**), and 9-OxoODE (**70**) were correlated with FRAP and TPC. Hydroxyglycitein (**41**) correlated only with FRAP. Previously, Lee *et al*. [11] reported that the antioxidant activity of hydroxy-isoflavonoids was superior to that of other isoflavonoid forms. As shown in Fig. 5, the TPC and FRAP showed a strong correlation between the bioactivity networks. According to previous studies, high total phenol content could be correlated to high reducing power, so it could be associated with the high total phenol content and FRAP activity of mushrooms [43]. Each analysis method related to antioxidant activities formed a correlation network related to each different compound. However, flavonoids, isoflavonoids, and soyasaponins, known for their contribution to the antioxidant capacity of fermented soy products, as well as several amino acids that are present at a high level in *meju* in group I, represented positive correlations with the TFC, TPC, and FRAP activity. Previous studies have reported that isoflavonoids exhibited around ten times higher antioxidant activities than did amino acids, but gochujang had several hundred times higher amino acid content than isoflavonoid content, and the amino acids affected its antioxidant activity in gochujang [21]. Moreover, Martínez‐Valverde *et al*. [44] found that the antioxidant activity of tomato extracts varied significantly with the tomato variety and the assay used. The correlation analysis revealed that the compounds are related to the bioactivities according to the proportions between the bioactivity value and the metabolite content in the sample. Furthermore, several non-identified compounds could be present among the main metabolites affecting the bioactivity in the correlation network. These results suggest that the structures of each food compound could influence the different activity assays in various ways.

## Conclusion

In this work, we performed the metabolite and microbial profiling of 11 meju samples. Surprisingly, the metabolite profiling revealed a trend of the samples being divided into two groups: an amino acid-abundant group and a secondary metabolite-abundant group, consisting of M1–5 and M6–11, respectively. The results of the correlation analysis showed that most of the secondary metabolites were ABTS related, whereas amino acids and certain flavonoids were rather FRAP related. Thus, we could conclude based on the present study that the metabolites contribute to the antioxidant capacity of the meju samples depending on the analytic method of the antioxidant activity. Future research should aim at understanding the importance of meju analysis. Therefore, we emphasize the importance of performing future research focusing on good quality meju-derived doenjang (thick soybean paste) and ganjang (soy sauce) metabolite analysis.

## Supplemental Materials



Supplementary data for this paper are available on-line only at http://jmb.or.kr.

## Figures and Tables

**Table 1:** General characteristics of 11 traditional meju samples.

Sample name	Region of Korea	NaCl (titration)	Meju weight (g)	Water (L)
M1	Jeollabukdo	18.2	9.0	20
M2	Gyeonggido	20.3	5.9	25
M3	Gyeongsangnamdo	21.7	4.8	20
M4	Gyeongsangbugdo	22.6	10.4	18
M5	Chungcheongbukdo	14.2	5.3	20
M6	Jeollabukdo	16.9	9.3	18
M7	Jeollabukdo	13.9	10.0	18
M8	Gyeongsangbugdo	13.2	9.0	20
M9	Chungcheongnamdo	16.8	5.9	20
M10	Chungcheongbukdo	13.6	5.8	27
M11	Jeollanamdo	14.3	5.6	20
