# Supplementary material for: Metabolite Profiling and Microbial Community of Traditional Meju Show Primary and Secondary Metabolite Differences Correlated with Antioxidant Activities
Source: J Microbiol Biotechnol. 2020 Sep 2;30(11):1697–705. doi: 10.4014/jmb.2007.07026 (PMC9728299; doi:10.4014/jmb.2007.07026)
Supplement: Supplementary file 1 [file JMB-30-11-1697-supple.pdf]

J. Microbiol. Biotechnol.
https://doi.org/10.4014/jmb.2007.07026

Supplementary data

Fig. S1. PCA score plots derived from non-targeted metabolite profiling of 11 *meju* types analyzed using GC–TOF-MS (A) and UHPLC–Orbitrap-MS/MS (B). PCA Score plots for *meju* samples: (■); M1 (■), M2 (■), M3 (■), M4 (■), M5 (■), M6 (■), M7 (■), M8 (■), M9 (■), M10 (■), M11 (■).

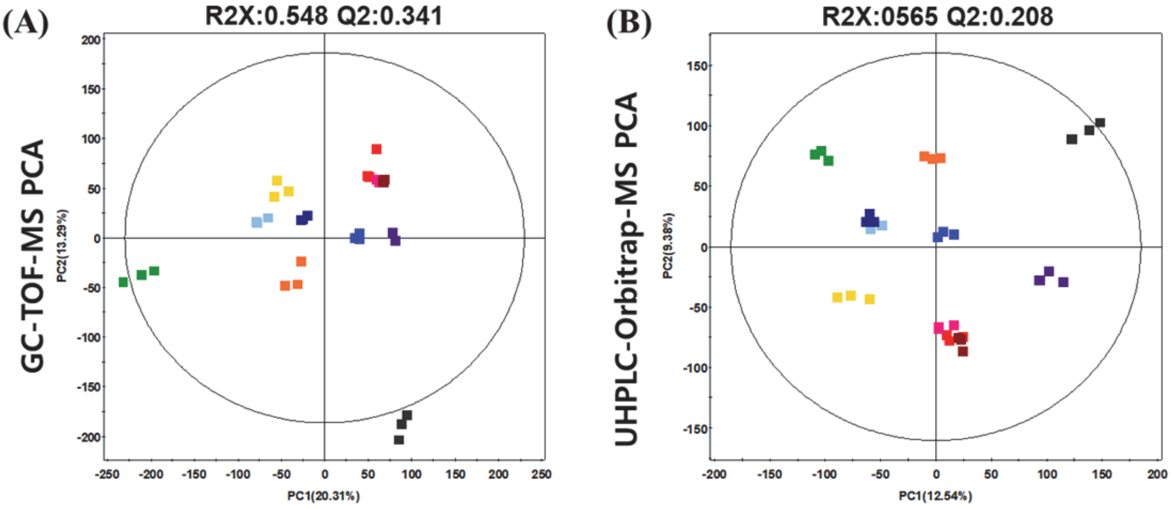

Table S1. Tentatively identified meju metabolites from different materials based on the GC-TOF-MS analysis.

		GC-TOF-MS			
**S.No.**	**Tentative identifications***	**RT** (min)**	**Identified ion (*m/z*)**	**Mass Fragment pattern (*m/z*)*****	**TMS****
Amino acids					
1	Valine	6.7	144	73 144 147 218 100 59 75 145 74	2
2	Leucine	7.2	158	158 73 102 159 100 59 75 74	2
3	Glycine	7.6	174	73 174 86 147 59 100 175 133	3
4	Serine	8.1	204	73 204 218 100 147 75 74	3
5	Threonine	8.3	57	73 57 117 101 219 218 147	3
6	Aspartic Acid	9.5	232	73 232 100 147 75 74 117 233	3
7	Phenylalanine	10.4	218	73 218 192 100 147 75 74 219	2
8	Ornithine	11.8	142	73 142 174 59 86 74 147 100 143	4
9	Lysine	12.5	156	73 156 174 59 86 128 100 74	4
10	Histidine	12.5	154	73 203 154 147 75 74 103 59	3
11	Tyrosine	12.6	100	73 218 100 147 75 219 74 103	3
12	Tryptophan	14.4	202	73 202 75 203 74 55 204 117 129	3
Fatty acids					
13	Butanoic Acid	8.7	75	73 147 75 101 189 117 74 133 59	3
14	Hexadecanoic Acid	13.2	132	117 75 73 132 129 55 145 131	1
15	Oleic Acid	14.2	98	75 117 73 55 129 145 81 96 67 84	1
16	Linolenic Acid	14.3	335	75 79 73 55 67 95 117 129 81 93	1
Sugar & sugar derivatives					
17	Glycerol	7.3	117	73 117 103 205 147 218 133	3
18	Pinitol	11.9	133	73 147 133 217 260 86 191 103 74 75	5
19	Adonitol	12.1	103	73 103 147 217 68 67 149 89 74 129	5
20	Glucosamine	13.6	202	73 147 129 87 75 117 202 74	4
21	Sucrose	16.7	361	73 147 103 217 361 129 169	7
22	Maltose	16.8	204	73 204 147 103 129 217 75 74	8
Etc					
23	Urea	6.9	189	147 73 189 171 66 148 74 99 59	2
24	Benzoic Acid	7.0	105	105 77 179 165 51 180 50	1
Non-Identifications					
25	N.I. 1	9.4	103	147 103 117 133 59 129 11 148	4

26	N.I. 2	9.9	129	73 129 147 75 85 157 247 133	3
27	N.I. 3	14.2	262	75 73 55 67 81 129 79 95 117	1
28	N.I. 4	15.3	331	73 75 55 67 81 129 131 117	2
29	N.I. 5	17.2	361	73 191 147 361 103 129 217	7

*Tentative metabolites based on VIP>1.0 and $p<0.05$ based on PLS-DA and one-way ANOVA analysis, respectively.

**RT, and TMS indicates retention time, and trimethylsilyl, respectively.

Table S2. Tentatively identified meju metabolites from different materials based on the UHPLC-Orbitrap-MS/MS analysis.

		UHPLC-Orbitrap-MS/MS							
S. No.	Tentative identifications*	RT** (min)	[M-H] -	[M+H] +	MW**	Elemental composition [M+H]+	Error (ppm)	MS^n fragment pattern (*m/z*)	ID**
Isoflavonoids									
30	Daidzin	4.51	415.1034	417.1169	416	C21H21O9	-2.778	417>287,269,255>254,227,211	Ref [1, 2]
31	Glycitin	4.61	445.1140	447.1277	446	C22H23O10	-1.841	447>429,316,285>270,229	Ref [1, 2]
32	Genistin	4.95	431.0980	433.1116	432	C21H21O10	-3.055	433>415,313,271>253,243,215,152	Ref [1, 2]
33	Malonyldaidzin	4.95	501.1977	503.1169	502	C24H23O12	-1.933	–	Ref [2]
34	Malonyglyictin	5.40	531.1149	533.1276	532	C25H25O13	-1.383	531>487,431,269>268,224,180	Ref [2]
35	Malonygenistin	5.33	517.1232	519.1120	518	C24H23O13	-2.537	519>433,271>270,242,214,148	Ref [2]
36	Acetyldaidzin	5.21	457.1138	459.1274	458	C23H23O10	-2.599	459>441,328,255,199	Ref [2]
37	Acetylglycitin	5.30	487.1777	489.1380	488	C24H25O11	-2.265	–	Ref [2]
38	Acetylgenistin	5.71	473.1079	475.1223	474	C23H23O11	-2.416	475>457,379,313,270>242,215,152	Ref [2]
39	Glycitein	5.87	283.0615	285.0750	284	C16H13O5	-2.491	285>285,270>252,242,214	Ref [1, 2]
40	Genistein	6.38	269.0446	271.0591	270	C15H11O5	-3.652	271>253,247,243>156,129	Ref [1, 2]
41	Hydroxyglycitein	6.29	301.0701	299.0563	300	C16H13O6	-1.975	–	Ref [1]
42	Hydroxygenistein	5.77	285.0405	287.0543	286	C15H11O6	-2.838	285>269,257,241>228,218	Ref [1]
Soyasaponin									
43	Soyasaponin A2	5.43	1105.5431	1107.5563	1106	C53H87O24	-2.826	1107>945,597,421>239,227,184	HMDB***
44	Soyasaponin Bf	6.47	925.4783	927.4949	926	C47H75O18	-1.554	925>907,717,>567,511,471	Ref [3]
45	Soyasaponin Aa	6.44	1363.6152	1365.6313	1364	C64H99O31	-1.723	–	Ref [4]
46	Soyasaponin Ab	6.54	1435.6367	1437.6517	1466	C67H105O33	-1.828	–	Ref [4]
47	Soyasaponin Ae	6.68	1201.5640	1203.5775	1202	C58H91O26	-1.495	1201>1159,967,719,605,473	Ref [4]
48	Soyasaponin Ag	6.75	1171.5514	1173.5680	1172	C57H89O25	-0.643	1171>1129,833,719,605>473	Ref [4]
49	Soyasaponin Af	6.79	1273.5848	1275.6003	1274	C61H95O28	-0.077	1273>1231,1043,749,605>473	Ref [4]
50	Soyasaponin Ah	6.82	1243.5732	1245.5870	1244	C60H93O27	2.440	1243>1231,1043,749,605	Ref [4]
51	Soyasaponin I	7.05	941.5108	943.5250	942	C48H79O18	-1.200	943>325,797,599>581,423,351	Ref [2, 5]
52	Soyasaponin II	7.26	911.4996	913.5126	912	C47H77O17	-3.171	911>893,765,457>437,371	Ref [2, 5]
53	Soyasaponin III	7.29	795.4519	797.4656	796	C42H69O14	-3.264	797>779,599,423,365>203	Ref [2, 5]
54	Soyasaponin IV	7.39	765.4418	767.4562	766	C41H67O13	-1.874	765>721,615,457	Ref [2, 5]
Glycerophospholipids									
55	LysoPC18:3	8.12	562.3145[a]	518.3217	517	C26H49NO7P	-4.622	518>500,258,184>162	Ref [5]
56	LysoPC18:2	8.51	518.2885	520.3381	519	C26H51NO7P	-3.490	520>502,443,397,337>323	Ref [5]
57	LysoPC16:0	8.81	540.3309[a]	496.3376	497	C24H51NO7P	-4.323	496>478,466,421>419,103	Ref [5]

58	LysoPC18:1	9.04	566.3459[a]	522.3529	521	C26H53NO7P	-4.797	522>504,445>419,309	Ref [5]
59	LysoPC18:0	9.73	568.3617[a]	524.3691	523	C26H55NO7P	-3.787	524>506,447,341>311	Ref [5]
Flavonoids									
60	Naringenin-7-O-glucoside	3.89	433.1143	435.117	434	C21H23O10	-1.547	433>415,271,205>150	Ref [6]
61	Naringenin	6.38	271.0611	273.0645	272	(-) C15H11O5	-0.246	–	Ref [6]
62	Luteolin 7-rutinoside	4.64	609.1449	611.1585	610	(-) C27H29O16	-1.983	609>591,429,285,255,179	Ref [4]
63	Luteolin 7-methyl ether	5.23	299.0561	301.0699	300	C16H13O6	-2.274	299>284,253>227,184	Ref [4]
64	Luteolin	5.49	285.0406	287.0542	286	C15H11O6	-2.733	285>256,241>213	Ref [4]
Oxylipins									
65	9,12,13-TriHOME	6.54	329.2329	331.1872	330	(-) C18H33O5	-1.420	329>311,293>185,171	Ref [7]
66	9,10-DiHODE	7.12	311.2223	313.2362	312	(-) C18H31O4	-1.583	311>293,275,255,157	Ref [7]
67	9(S)-HpODE	7.66	311.2225	313.2368	312	(-) C18H33O4	-3.712	311>293,281>275,249,191,139	Ref [7]
68	12,13-DiHOME	8.12	313.2375	315.2516	314	(-) C18H33O4	-2.818	313>295,277>195,183	Ref [7]
69	13-HODE	8.96	295.2275	297.2613	296	(-) C18H31O3	-1.111	295>277>275,259,233	Ref [7]
70	9-OxoODE	9.20	293.2124	295.2257	294	(-) C18H29O3	0.416	293>275,265>257,255	HMDB
Non-identification									
71	N.I. 6	5.97	1089.5463	1091.5616	1090	–	–	1091>929,731,581,423,365>203	–
72	N.I. 7	6.04	1073.5524	1075.5670	1074	–	–	1073>1055,747,589>367	–
73	N.I. 8	8.29	416.2909	418.3046	417	–	–	418>400,372>355,243	–
74	N.I. 9	9.14	426.2683	428.2812	427	–	–	428>410,364>318,263	–
75	N.I. 10	9.37	408.2761	410.2885	409	–	–	410>392,263>129	–
76	N.I. 11	9.50	438.3329	440.3465	439	–	–	438>421,395>377,351,131	–
77	N.I. 12	9.67	380.2808	382.2939	381	–	–	382>264,336>318,263	–
78	N.I. 13	10.50	378.3006	380.3141	379	–	–	380>362,334,263>261,184	–

*Tentative metabolites based on VIP>1.0 and $p<0.05$ based on PLS-DA and one-way ANOVA analysis, respectively.

**RT, MW, ID, Ref, and HMDB indicates retention time, molecular weight, identification, reference, and https://hmdb.ca/, respectively.

***superscript ‘a’ indicates the $[M\text{-}FA+H]^-$.

Supplementary references

1. Lee S, Seo M-H, Oh D-K, Lee CH. 2014. Targeted metabolomics for Aspergillus oryzae-mediated biotransformation of soybean isoflavones, showing variations in primary metabolites. *Biosci. Biotech. Bioch.* **78:** 167-174.
2. Lee SY, Lee S, Lee S, Oh JY, Jeon EJ, Ryu HS*, et al.* 2014. Primary and secondary metabolite profiling of doenjang, a fermented soybean paste during industrial processing. *Food Chem.* **165:** 157-166.
3. Lee S-Y, Kim J-S, Shim S-H, Kang S-S. 2011. Soyasaponins from Soybean Flour Medium for the Liquid Culture of Ganoderma applanatum. *B Korean Chem Soc.* **32:** 3650-3654.
4. Suh DH, Jung ES, Park HM, Kim SH, Lee S, Jo YH*, et al.* 2016. Comparison of metabolites variation and antiobesity effects of fermented versus nonfermented mixtures of Cudrania tricuspidata, Lonicera caerulea, and soybean according to fermentation in vitro and in vivo. *PLoS One.* **11**.
5. Kwon YS, Lee S, Lee SH, Kim HJ, Lee CH. 2019. Comparative Evaluation of Six Traditional Fermented Soybean Products in East Asia: A Metabolomics Approach. *Metabolites.* **9:** 183.
6. Zeng X, Su W, Zheng Y, Liu H, Li P, Zhang W*, et al.* 2018. UFLC-Q-TOF-MS/MS-based screening and identification of flavonoids and derived metabolites in human urine after oral administration of Exocarpium Citri Grandis extract. *Molecules.* **23:** 895.
7. Strassburg K, Huijbrechts AM, Kortekaas KA, Lindeman JH, Pedersen TL, Dane A*, et al.* 2012. Quantitative profiling of oxylipins through comprehensive LC-MS/MS analysis: application in cardiac surgery. *Anal Bional Chem.* **404:** 1413-1426.
